# High-Pressure Sorption of Hydrogen in Urea

**DOI:** 10.1021/acs.jpcc.1c00138

**Published:** 2021-03-31

**Authors:** F. Safari, M. Tkacz, A. Katrusiak

**Affiliations:** † Faculty of Chemistry, Adam Mickiewicz University, ul. Uniwersytetu Poznańskiego 8, 61-614 Poznań, Poland; ‡ Institute of Physical Chemistry PAS, Kasprzaka 44/52, 01-224 Warszawa, Poland

## Abstract

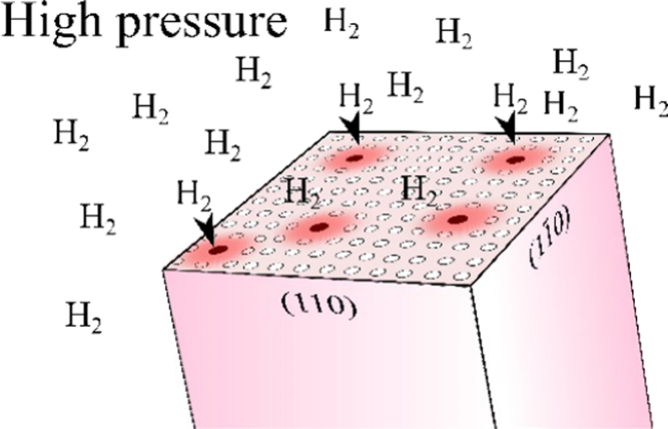

Hydrogen sorption in urea C(NH_2_)_2_O has been probed by direct measurements in Sievert’s apparatus at 7.23 and 11.12 MPa as well as by Raman spectroscopy for the sample compressed and heated in a high-pressure gas-loaded diamond-anvil cell up to 14 GPa. Both these methods consistently indicate the occurrence of small nonstoichiometric sorption of hydrogen in urea phase I. The compression of urea in hydrogen affects the Raman shifts of the C–N bending mode δ and the stretching mode υ_s_. The sorption affects the H_2_ vibron position too. The sorption of 1.3 × 10^–2^ at 11.12 MPa corresponds to a stochastic distribution of H_2_ molecules in channel pores of urea. The mechanism leading to this stochastic sorption involves strong correlations between the swollen nanodot regions around the pores accommodating H_2_ molecules and the squeezed neighboring pores too narrow to act as possible sorption sites. This study on the hydrogen-bonded framework (HOF) of urea marks the smallest pores capable of absorbing hydrogen documented so far. This observation also reveals a new class of compounds, which is located between those that absorb large stoichiometric amounts of certain guest molecules and those that do not absorb them at all, namely, the group of compounds that absorb the guests in a stochastic manner.

## Introduction

The application of hydrogen fuels has become an important task aimed at environment-friendly technologies. Because of the relatively high compression required for the H_2_ storage, new porous materials capable of clathrating H_2_ molecules in their structure are intensely investigated. The urea crystals are well known for their structure containing small pores,^1−4^ and it was postulated that this compound could be used for H_2_ storage.^5,6^ This opinion was based on the size of channel pores in the urea crystal and on the dimensions of the H_2_ molecule, as the main parameters connected to the clathrating capability. The application of urea was considered advantageous due to its low cost compared to other materials, which are used in the industry for the storage of hydrogen.^6^

**Figure 1:**
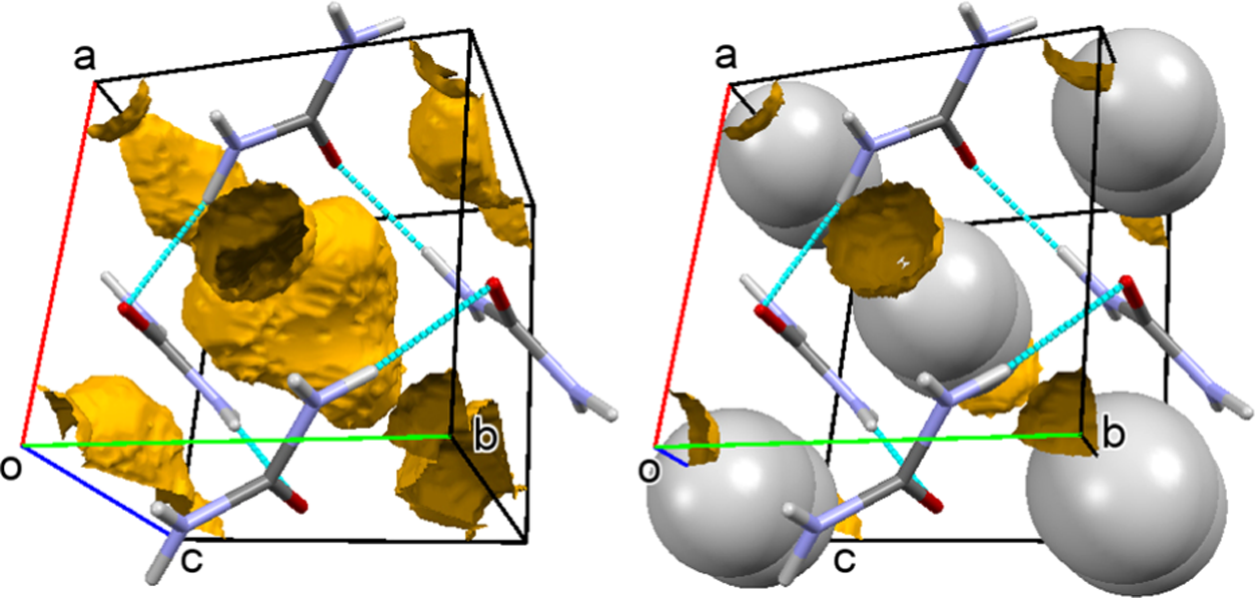
Projections of the urea phase I structure (capped stick model and the NH···O bonds marked by a blue dashed lines): (left) with the voids (calculated with a probe radius of 0.6 Å and a grid spacing of 0.1 Å) marked in yellow^7^ and (right) the same structure with the largest portions of the pores filled with H_2_ molecules shown as van der Waals spheres.

The pores in urea, of about 2 Å in diameter (Figure 1), are significantly smaller than the van der Waals dimension of the H_2_ molecule perpendicular to its H–H bond of about 2.4 Å according to Bondi^8^ or 2.2 Å according to Rowland and Taylor.^9^ It was suggested that in H_2_ molecules, the van der Waals radius of H atoms increases,^10^ but this information was not confirmed.^9^ On the other hand, it was demonstrated that intermolecular contacts, which are often used for assessing the van der Waals radii, are considerably reduced under high pressure. For example, in the ferrocene crystals, the shortest intermolecular H···H distance is reduced from 2.599 Å at 0.1 MPa to 2.151 Å at 2.89 GPa;^11^ in the urea crystal, the shortest H···H contact is reduced from 2.766 to 2.118 Å between 0.1 MPa and 2.75 GPa.^12,13^ On average, the shortest H···H contacts in molecular crystals display the compressibility (β_d_ = −1/*d*·∂*d*/∂*p*) equal to 0.068 GPa^–1^. This value has been based on the structural data of hydrostatically compressed crystals of organic compounds between 0.1 MPa and 0.5 GPa. Presently, we have investigated the consequences of the van der Waals radii compression for the voids in the urea structure as well as their accessibility at high pressure when all dimensions of the urea and H_2_ molecules are squeezed.

The compression of the van der Waals radii strongly favors the penetration of potential molecular guests because the radii of atoms are compressed, as are the radii of atoms on the walls of the pores. The difference (Δ) between the pore diameter (*D*) and the diameter of the guest (*G* = 2*R*_vdW_) is
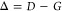
1

The negative values of Δ indicate that the diameter of the guest molecule is larger than the pore diameter. After increasing pressure (*p*), the van der Waals radii of atoms are compressed at the rate approximately equal to half of β_d_. This pressure effect can be included in eq 1 rewritten in the form
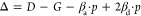
2where β_a_ is the linear compression of the crystal along the [*x*] direction (in the case of tetragonal urea phase I, along any direction perpendicular to [*z*]). Most importantly, eqs 1 and 2 describe the average crystal structure, where all unit cells are identical. Donnelly et al. investigated the sorption of D_2_ in the deuterated urea C(ND_2_)_2_O powder by neutron diffraction.^14^ They performed careful measurements within the region of urea phase I and at the higher-pressure region of phase III. According to their measurements of the lattice dimensions, molecules D_2_ do not penetrate the C(ND_2_)_2_O structure in all investigated high-pressure range up to 3.7 GPa.^14^

**Figure 2:**
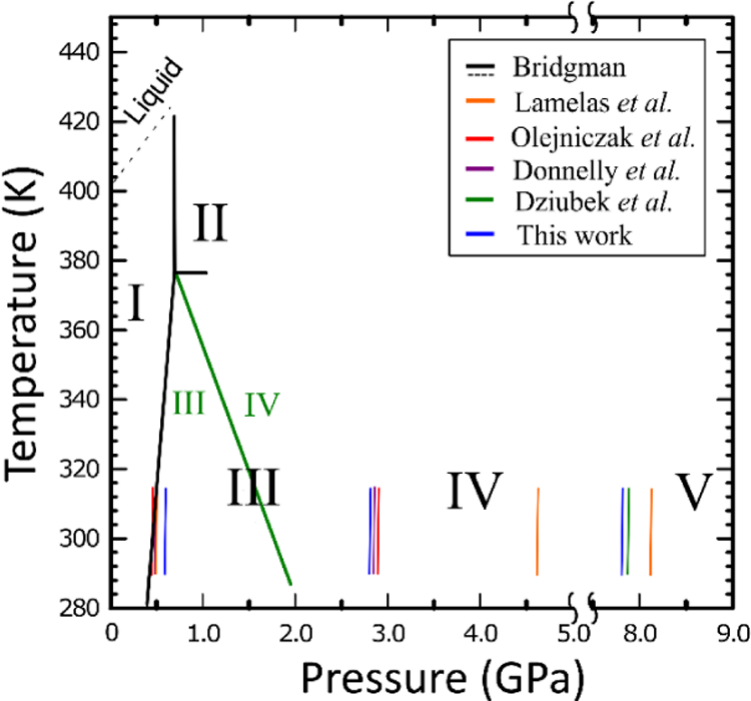
Phase diagram of urea based on this work and the literature.^12−14,18−21^ The vertical lines indicate the phase boundaries determined at room temperature in different studies (see the legend) and in different pressure-transmitting media. Phase II postulated by Bridgman^19^ was not confirmed in other studies^13,21^ (see the text).

The urea crystals belong to the most thoroughly investigated organic materials,^15−17^ and it was established that they do not undergo solid–solid phase transitions induced by temperature changes.^18^ However, pressure-induced phase transitions in urea were detected by volumetric studies by Bridgman,^19^ who postulated that above 370 K and 0.5 GPa, phase I transforms to phase II, and that at 293 K and 0.48 GPa, phase I transforms to phase III at room temperature; he also mapped the melting curve of urea as a function of pressure. It was later established by X-ray diffraction that at 0.48 GPa, the pores of urea phase I collapse when the crystal transforms into phase III,^12,13^ and that the pores remain closed even tighter in phase IV above 2.80 GPa.^12,13,20^ Further studies revealed yet another closely packed phase V above 7.0 GPa (Figure 2, cf. Table S1).^18,20^ The existence of phase II was questioned later when it was attempted to reach it in a high-temperature high-pressure single-crystal study,^13^ and other high-temperature high-pressure powder synchrotron X-ray diffraction and FTIR absorption studies^21^ indicated that phase IV extends to the area suggested for II by Bridgman.^19^ Nonetheless, the original labels of urea phases introduced by Bridgman^19^ have been adopted throughout this work.

## Experimental Section

For our Raman measurement, we used a diamond-anvil cell (DAC) equipped with the type II, low-fluorescence diamonds with the culet diameter of 400 μm. A rhenium gasket was prepared by preindenting the foil to the thickness of about 50 μm and laser-drilling a hole of 200 μm in diameter. The pressure in the DAC chamber was measured by the ruby fluorescence method.^22^ The Raman spectra were recorded by a THR1000 spectrometer with a He–Ne laser line (excitation 632.8 nm). In the first series of experiments, hydrogen was loaded into the DAC chamber partly filled with a fine urea powder at the initial pressure of about 0.2 GPa by the gas-loading technique described earlier.^23^ In the DAC chamber, hydrogen was in significant excess (in the molar ratio and volume) over urea in all experiments, which secured the hydrostatic conditions for the urea sample. Then, the pressure was increased in steps, and the Raman spectra were recorded for each pressure up to 14 GPa. In the second series of measurements after loading hydrogen at 0.2 GPa, the pressure was increased in small steps to 0.36 GPa, and the DAC was heated to 373 K for about 1 h. The Raman spectra were recorded after cooling the DAC to room temperature at 0.4, 0.53, 0.69, 0.96, 1.26, and 1.55 GPa. In another experiment, the DAC was filled with the urea powder and immersion oil as the pressure-transmitting medium, and Raman spectra were recorded up to 4.7 GPa.

We also performed the hydrogen sorption measurement on the urea grounded powder of few micron-size grains subjected to gaseous hydrogen in the homemade Sievert’s-type apparatus.^24^ Hydrogen gas pressure was measured by a piezoelectric gauge covering pressure range up to 20 MPa with 0.0001 MPa resolution connected to a pressure monitor Druck DPI-145. Two runs of the sorption and desorption experiments, one at 7.23 MPa and the other at 11.12 MPa, were carried out on the samples of 13.005 and 8.126 g of powdered urea, respectively. After pressurizing the urea sample in hydrogen, its pressure was monitored as a function of time. After about 12 h, the pressure was released to 0.1 MPa, the system was sealed, and the pressure was monitored as a function of time again. For the experiment at 7.23 MPa, the characteristic of reversible sorption and desorption time evolution was observed. However, for the investigation at 11.12 MPa, the desorption was much slower compared to the quicker and stronger sorption process, which is an indication of different mechanisms of the sorption of H_2_ in different pressure ranges.

## Results and Discussion

It is well known that high pressure promotes the formation of clathrate compounds. For example, in arsenolite, despite the absence of pores connecting the voids, they are filled with He atoms above 3 GPa. The sorption of He proceeds from the surface of the crystal, and the layer of the As_4_O_6_·2He clathrate becomes deeper with time.^25,26^ This kinetic process requires hours for the He atoms to penetrate few microns below the surface of arsenolite crystals. Hydrogen forms the smallest diatomic molecule and it readily diffuses through most liquids and many solids.^27−29^

The voids in the urea crystal are only slightly smaller than the H_2_ molecule and the voids are connected into channels, hence the assumption that the H_2_ molecules can be absorbed at high pressure.^5,6^ However, the high-pressure neutron-diffraction studies on deuterated urea detected no formation of its inclusion compound with D_2_ up to 3.7 GPa.^14^ In our present study, we focused on the possibility of nonstoichiometric sorption of hydrogen in urea, which does not manifest in the average crystal structure transformations. For these type of investigations, we have used Raman spectroscopy and direct measurement of the compressed H_2_ gas sorption in urea using highly accurate Sievert’s apparatus.

The Raman spectra of the urea powder compressed in H_2_ in a DAC contained three anomalies in the shifts, which mark the structural transitions in urea at room temperature between ambient-pressure phase I stable up to 0.48 GPa (tetragonal, with two molecules per unit cell, space group *P*4̅2_1_*m*), phase III stable up to 2.8 GPa (orthorhombic, four molecules per unit cell, space group *P*2_1_2_1_2_1_), phase IV stable up to 7.2 GPa (*P*2_1_2_1_2),^12^ and phase V (*Pmcn*) stable at still higher pressure.^18^ The compression of urea in the oil induces the transition between phases I and III at 0.48 GPa, in accordance with the previous high-pressure experiments.

However, the compression of the urea powder in hydrogen induces this transition at somewhat higher pressure at 0.53 GPa. This systematic increase in the *p*_13_ value for several experiments with H_2_, compared to *p*_13_ = 0.48 GPa for the compression of urea in other media, was an indication that the H_2_ molecules can penetrate the pores and support their walls under pressure. The *T*–*p* phase diagram of urea was investigated by several techniques.^12−18^ The reported phase diagrams are compiled in the Supporting Information, and some of them have been plotted together with the results of our determination of *p*_13_, as shown in Figure 2.

**Figure 3:**
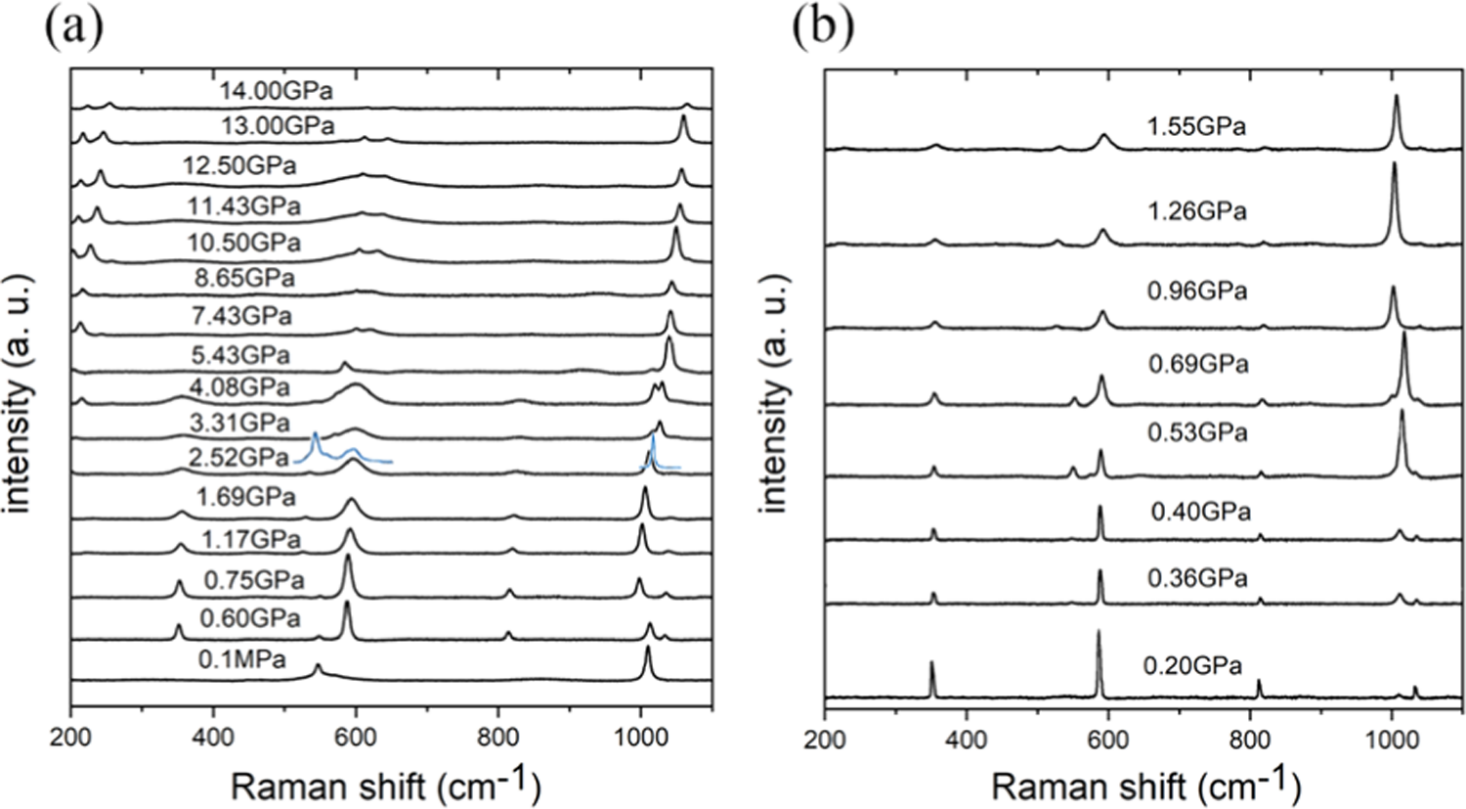
(a) Selected Raman spectra of urea at 0.1 MPa and urea–hydrogen up to 14.0 GPa and blue spectra are from Lamelas et al. at 2.9 GPa.^20^ (b) Second experiment up to 1.55 GPa; the intensities of spectra are normalized to allow comparison of all modes.

**Figure 4:**
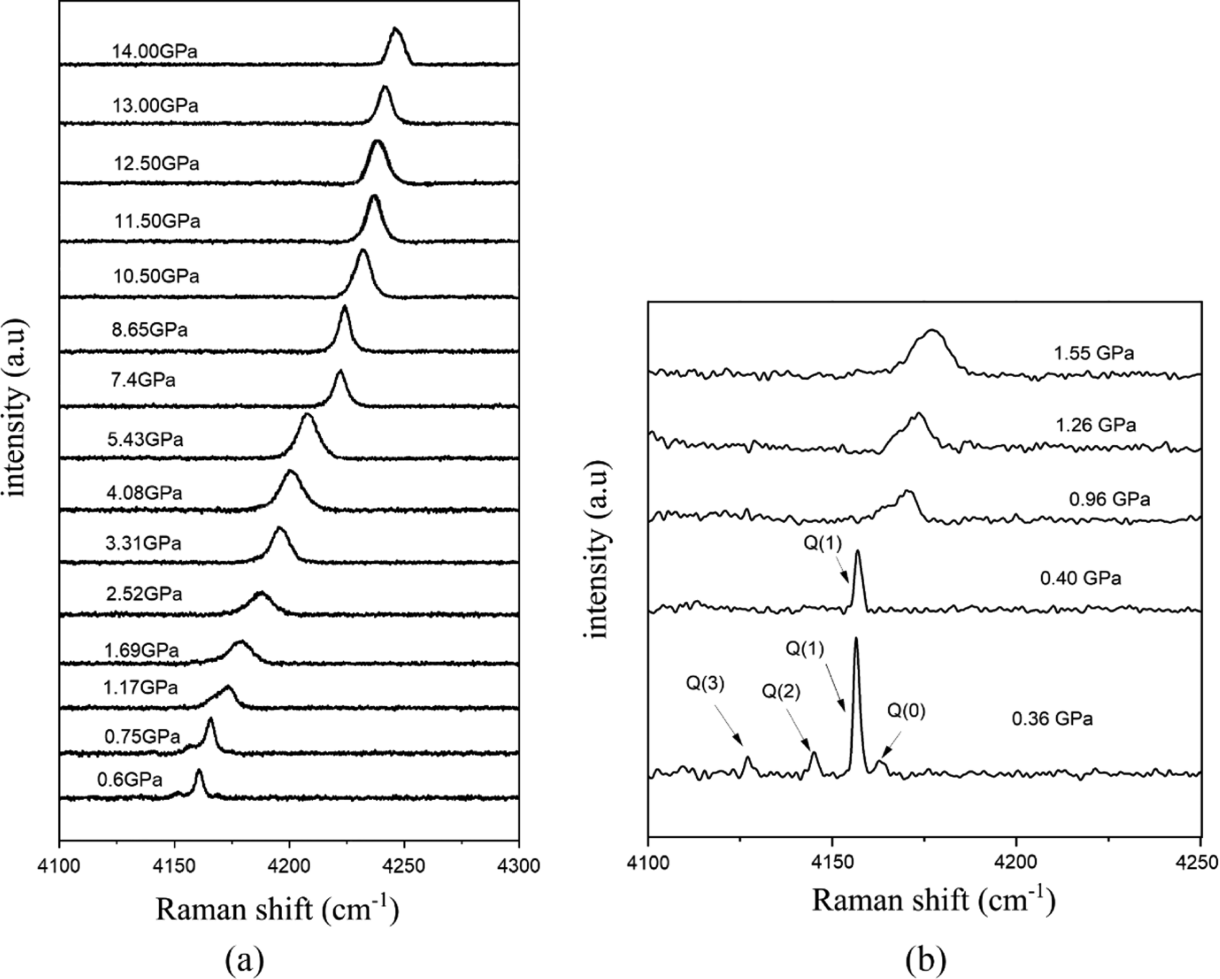
(a) Raman spectra of hydrogen vibration (Q_1_-branch) up to 14.0 GPa. (b) Second series of experiments, with vibration modes of hydrogen indicated.

Raman spectroscopy is a sensitive method for investigating the sorption because the presence of guest molecules in the channel pores affects both the bending and stretching modes of urea due to its strong intermolecular interactions with the guests, which can exert some strain on the molecules and restrict their vibrations in the lattice. The Raman spectra of urea at ambient and high-pressure conditions when compressed in Ar gas were measured by Lamelas et al.,^20^ and their results well agree with our measurements performed for the urea powder compressed in oil. At the ambient conditions, the C–N bending mode δ(CN) with the Mulliken symbol A_1_ occurs around 550 cm^–1^ and the C–N stretching mode υ_s_(CN) with A_1_ symmetry near 1020 cm^–1^. For the pure urea sample, the bending-mode band splits into a doublet at 0.48 GPa on the transformation to phase III, when the channel pores collapse and one molecule becomes symmetry independent in the general position (in space group *P*2_1_2_1_2_1_), contrasted to the special positions of point-group symmetry *C*_2v_ of molecules in phase I (space-group symmetry *P*4̅2_1_*m*). The Raman frequencies significantly shift for the urea sample compressed in H_2_ (Figure 3, cf. Figure S7). For the first spectrum recorded immediately after loading the H_2_ gas into the DAC chamber, the bending mode of urea is blue-shifted by 20 cm^–1^ and the stretching mode band significantly reduces its intensity. These changes can be due to the presence of hydrogen in channel pores because the H_2_ molecules exert pressure on the urea molecules around them (in the walls of the channel pores) and significantly increase the force constants for their vibrations (Figure 3). As expected, the S_0_-branch of hydrogen at 557 cm^–1^ overlaps with the bending mode of urea. Moulton et al.^30^ showed that the vibrational modes of hydrogen are shifted up to 4.6 GPa. However, according to our spectra, the vibration modes belong to Q_1_(2), and Q_1_(3)-branch disappeared when hydrogen molecules entered the urea channel pores above 0.36 GPa (Figure 4b), which can be due to interactions between hydrogen and urea molecules. Above 0.53 GPa, at 0.60 and 0.75 GPa in Figure 4a, when the crystal is transformed to phase III, the vibrational mode Q_1_(1) of hydrogen splits into two peaks due to the collapse of channel pores and extrusion of some of absorbed H_2_ molecules off the crystal. Consequently, for the H_2_ molecules inside and outside the crystal, the vibrational mode Q_1_(1) broadens in the pressure range of phase III due to the effects of pressure on interactions and collisions of H_2_ molecules. At still higher pressure of 1.17 GPa, these two peaks overlap, resulting in an asymmetric band.

**Figure 5:**
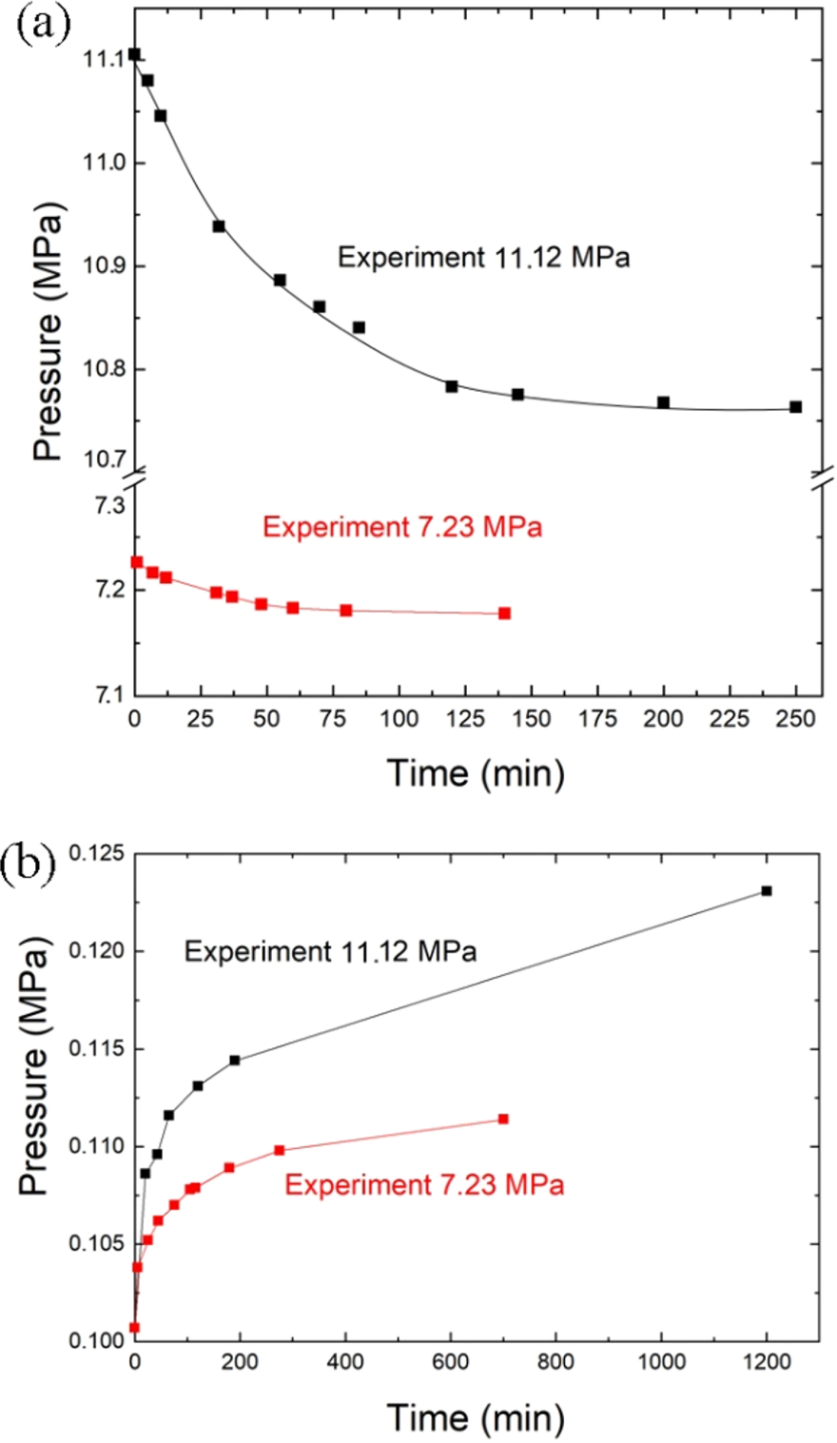
(a) Sorption and (b) desorption plots for the urea compressed in hydrogen at 296 K in Sievert’s apparatus for the experiments at 11.12 MPa (black plots) and 7.23 MPa (red).

The experiments in Sievert’s apparatus revealed small but significant sorption of H_2_ in urea crystals (13 g) immediately after increasing pressure to 7.23 MPa after connecting the sample to the reference chamber, and then gradual slower sorption was observed on saturating for about 120 min (Figure 5). On releasing pressure to 0.1 MPa and sealing the sorption again, the pressure increased in the characteristic manner of desorption to 0.1065 MPa after 60 min and to 0.1070 MPa after 80 min. The Sievert method experiments at 7.23 MPa show that the sorption of H_2_ in urea is slow and in a small nonstoichiometric ratio of 4.5 × 10^–4^ H_2_ per mol. However, both the sorption and desorption experiments consistently indicate similar kinetics resulting from the reversible character of the sorption.

Lamelas et al.^20^ reported that for urea compressed in argon, the anomalous behavior of lattice-mode bands E, A_1_ and B_1_ blue-shifted from 174 cm^–1^ at 0.1 MPa in phase I to above 200 cm^–1^, accessible in our experiments above 4 GPa in the region of phase IV (Figure 3). Lamelas et al. observed a reduction in the intensity of these bands around 6 GPa, which they associated with a transition between phases IIIA and IIIB (according to Bridgman’s labels used in this paper, within the region of phase IV). They extensively discussed this behavior and presented several possible models for it, such as the presence of small orientational domains or ferroelectric-like effects and strong back-scattering on the polarized microregions.^20^ Our spectra (Figure 3) confirm the substantial reduction of these bands at 5.43 GPa.

**Figure 6:**
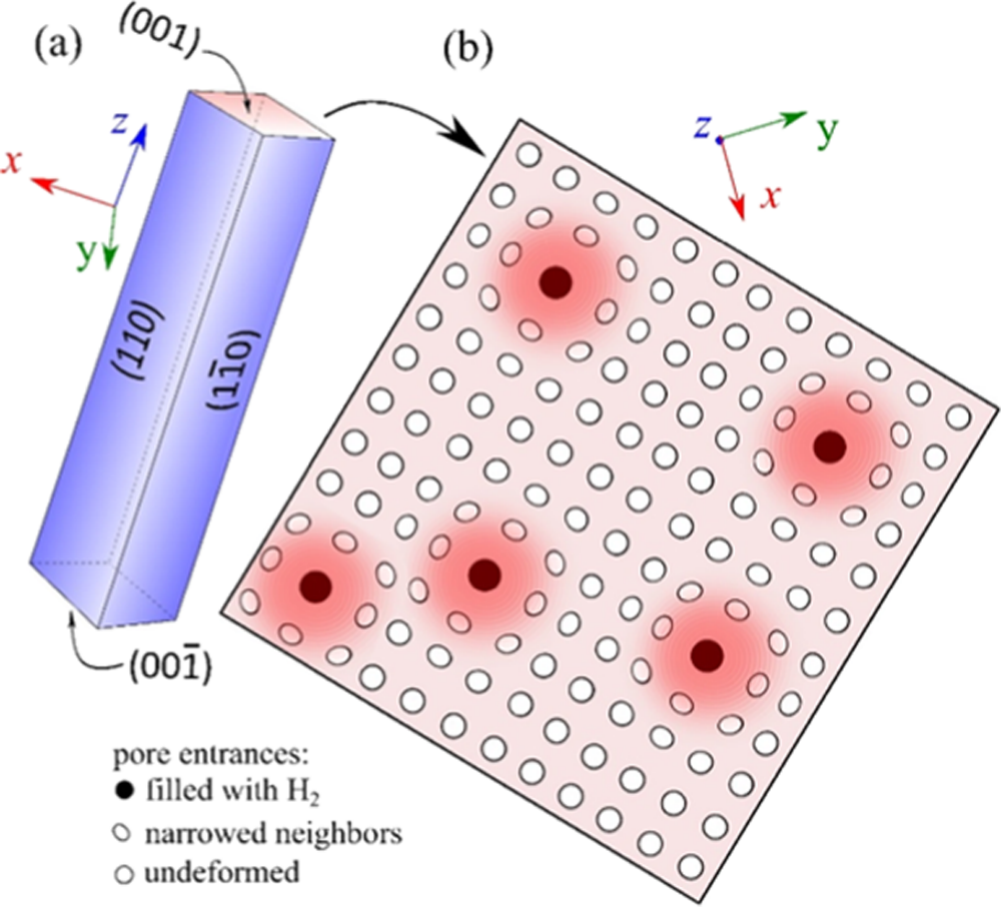
(a) Urea crystal habit of needle-like prisms elongated along the [*z*] direction, where entrances to the pores are located on small faces (001) and (001̅); (b) schematic illustration of the (001) face with the pattern of pores: stretched entrances of the pores filled with H_2_ guests, narrowed entrances of the neighboring empty pores, and the pores which are not deformed further away. The strain exerted by the pores occupied with H_2_ molecules is marked in the shades of red.

The results of our experiments show that the adsorbed H_2_ molecules enter into a small fraction of the voids in crystalline urea. In phase I, there are two urea molecules per each void, so the ratio of occupied to unoccupied voids at 7.23 MPa is 1:4 × 10^3^. When assuming a uniform distribution of H_2_ guests in the bulk of urea crystals, then this ratio would on average correspond to one H_2_ guest per crystal part of 16 × 16 × 16 unit cells. However, the urea crystals are strongly anisotropic, and the pores extend along the [*z*] direction. The crystals are in the form of strongly elongated needle-like prisms (Figure 6) with faces (001) and (001̅), containing the entrances of pores, much smaller than other faces. Consequently, H_2_ molecules can be absorbed only through the small faces (001) and (001̅), when the gas pressure increases.

**Figure 7:**
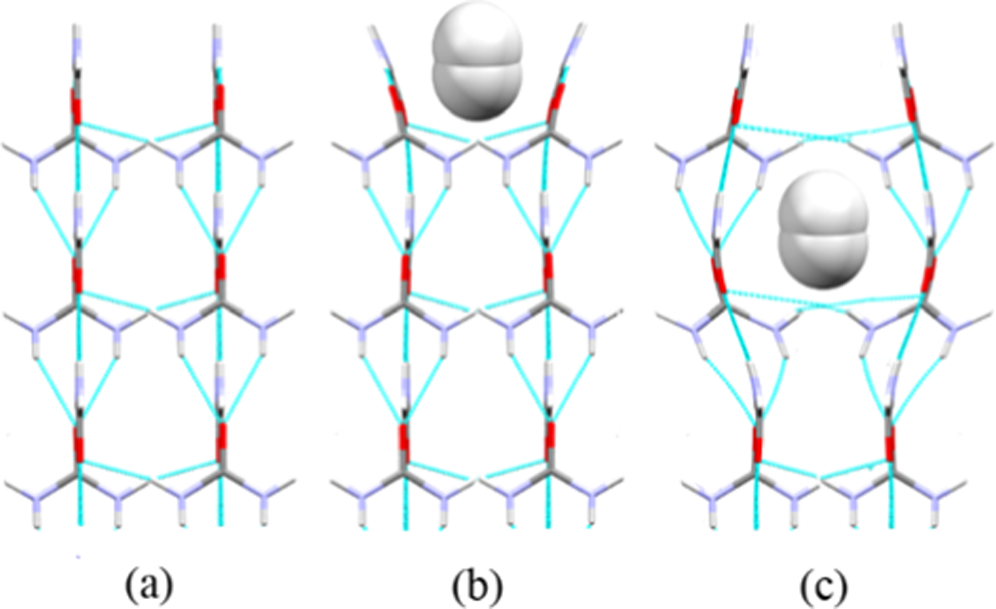
(a) Undisturbed channel pore of urea polymorph I (capped stick model), (b) H_2_ molecule (the space-filling model) entering pore, and (c) H_2_ molecule in the stretched urea pore expanded sideways to the neighboring pores.

It is possible that under this pressure, H_2_ molecules enter the pores stochastically on the surface, and that they expand the diameter of the pores by stretching the NH···O bonds (Figure 7). Due to this stretching, the neighboring pores are narrowed and they become closed for the entrance by other molecules. The required increase of the pore diameter is about 0.4 Å (eq 2), so the closest neighboring pores become by ca. 0.2 Å narrower. This strain can propagate further and it can cause narrowing of the next closest pores too, which has been illustrated in the schematic in Figure 6.

The Raman spectra, clearly different for urea crystals compressed in hydrogen from these compressed in other media, suggest that at high pressure, the H_2_ molecules penetrate further down the pores toward the bulk of the crystal grains. The lowest pressure of loading the DAC with hydrogen was 200 MPa (i.e., 0.2 GPa), hence considerably higher than the maximum pressure of 11.12 MPa in the sample chamber of Sievert’s apparatus. In another experiment with the Sievert’s apparatus started at 11.12 MPa, markedly stronger sorption of H_2_ was observed, but the desorption was only marginally, by about 10%, higher than that for the experiment at 7.23 MPa. We have observed that after the initial quick desorption, it continued at a slow rate even after several days. This result shows that the desorption process is much slower than sorption. The desorption proceeds gradually and the movements of guest H_2_ molecules are controlled by the diffusion along the pores and a small gradient in the interactions of H_2_ molecules with the walls of pores, gently pushing the molecules toward the crystal surface. This movement, apart from the tighter voids and the narrower sections of the pores, is additionally hindered by the correlation between the H_2_ movements within the range of interference through the strained lattice.

On releasing pressure, the strained lattice with the stretched NH···O bonds undoubtedly can reduce the internal energy by extruding the H_2_ guest molecules from the pores. It is also likely that the desorption of H_2_ guests occurs on increasing pressure to the *p*_13_ value. Owing to the presence of guest H_2_ molecules supporting the pore wall, the pressure of the transition to phase III is somewhat increased, but at 0.53 GPa, the H_2_ guests are released and the transition to phase III takes place. It is also possible that some of the H_2_ molecules are trapped in the collapsed structure, but their concentration is small and their distribution is stochastic so they do not affect the average structure of phase III.

**Figure 8:**
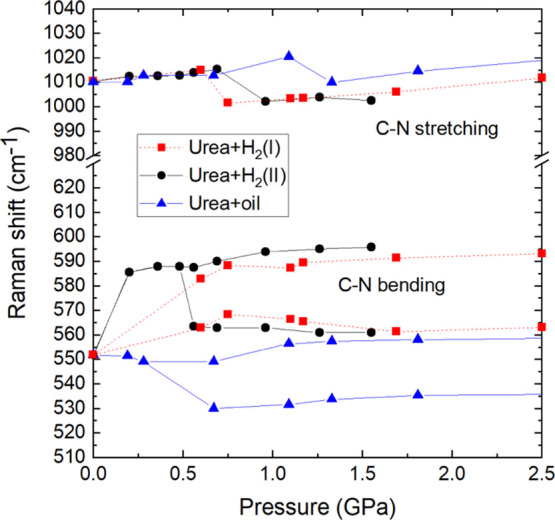
Raman shift frequencies as a function of pressure measured for the urea powder compressed in oil and in hydrogen (see the legend). The lines joining the points are for guiding the eye only. These plots in the pressure range extended to 5.0 GPa are shown in Figure S9.

Apart from a small increase in the critical pressure for the transition to phase III, we have also observed that the frequencies of CN bending modes of urea compressed in H_2_ clearly differ from those of urea compressed in oil (Figures 8 and S8). It is characteristic that the Raman shifts of urea compressed in H_2_ immediately after loading the DAC assume the frequency higher by about 45 cm^–1^ than the 0.1 MPa frequency, as well as those measured for urea compressed in oil. These differences continue into the regions of urea phases III and IV. For the stretching mode, there are no significant differences between the Raman shifts of urea compressed in oil and H_2_ in the pressure region of phase I, but there is a small difference of about 10 cm^–1^ in the region of phase III, and this difference disappears in the region of phase IV. The Raman shift difference for the stretching mode is opposite to that between the bending shifts.

## Conclusions

Our sorption and desorption experiments in Sievert’s apparatus measured up to 11.12 MPa as well as the Raman spectra recorded with the use of diamond-anvil cell up to 14.0 GPa reveals the stochastic type of sorption of H_2_ in the narrow channel pores of urea phase I. This stochastic sorption of H_2_ in urea is reversible on releasing pressure, although the kinetics of the desorption is much slower than that of the sorption. For this reason, it is likely that H_2_ molecules are only partly extruded from the pores when the urea crystals transform to phase III. The stochastic distribution of absorbed H_2_ molecules is due to the stretching of the pores, which in the average structure are somewhat too small to accommodate H_2_ molecules. Therefore, the stochastic sorption of H_2_ molecules requires that the neighboring pores absorb the strain by narrowing their diameter, which prevents the entrance of next H_2_ molecules around. The postulated stochastic H_2_ sorption mechanism is consistent with the results of previous neutron-diffraction studies on CO(ND_2_)_2_ urea compressed in D_2_^10^ in the respect that the stochastic sorption does not change the symmetry and average structures of phases I and III. The microscopic model of the H_2_ sorption assumes a differentiation of the structure into dot (0-D) as well as linear (1-D) clathrates, most likely concentrated close to the surface. It shows that sorption of gases can proceed according to different mechanisms, which can be described as the stoichiometric and stochastic sorption. For the stochastic sorption, the dimensions of the pores can be adjusted to those of guest molecules at the expense of the most immediate environment. It is possible that the stochastic sorption can be much stronger for other host compounds. On the one hand, it can be efficiently applied for detecting and separating different gases, as well as for other purposes, but on the other hand, it should be taken into account when the sorption is undesired or should be strictly prevented.

## Supporting Information Available

Raman shift frequency plots and phase diagrams of urea (PDF)
