# Supplementary material for: High-Pressure Sorption of Hydrogen in Urea
Source: J Phys Chem C Nanomater Interfaces. 2021 Mar 31;125(14):7756–62. doi: 10.1021/acs.jpcc.1c00138 (PMC8161694; doi:10.1021/acs.jpcc.1c00138)
Supplement: Supplementary file 1 — jp1c00138_si_001.pdf [file jp1c00138_si_001.pdf]

Supporting Information

High-Pressure Sorption of Hydrogen in Urea

F. Safari,[1] M. Tkacz,[2] A. Katrusiak[1]*

[1]Faculty of Chemistry, Adam Mickiewicz University, ul. Uniwersytetu Poznańskiego 8,

61-614 Poznań, Poland

[2]Institute of Physical Chemistry PAS, Kasprzaka 44/52, 01-224 Warszawa, Poland

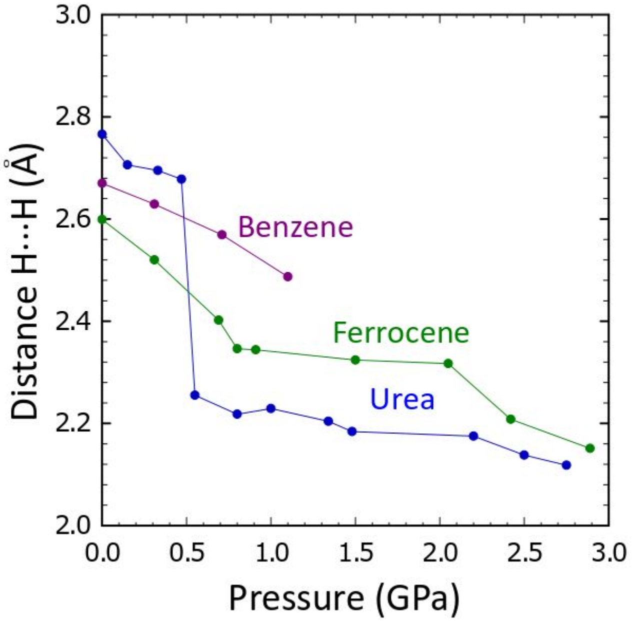

Figure S1 Shortest H···H bond in urea, ferrocene, and benzene up to 3.0 GPa.[1,2] The lines joining the points were drawn to guide the eye only.

Table 1. Phase transitions of urea in the literature.

References	*Transition*	*T (K)/P (GPa)*	*Space group*
Bridgman, P. W. (Proc. Am. Acad. Arts Sci. ***1916****)*	*I→II, III*	*0.48 GPa/296 K*	
Olejniczak, A. et.al (J. Phys. Chem. C ***2009****)*	*I→III*	*0.48 GPa/296 K*	$P\bar{4}2m \rightarrow P2_12_12_1$
	III→IV	*2.8 GPa/296 K*	$P2_12_12_1 \rightarrow P2_12_12$
Weber, H. P. et.al (J. Appl. Crystallogr. ***2002****)*	*IV →V*	*7.2 GPa/296 K*	$P2_12_12_1 \rightarrow Pmcn$

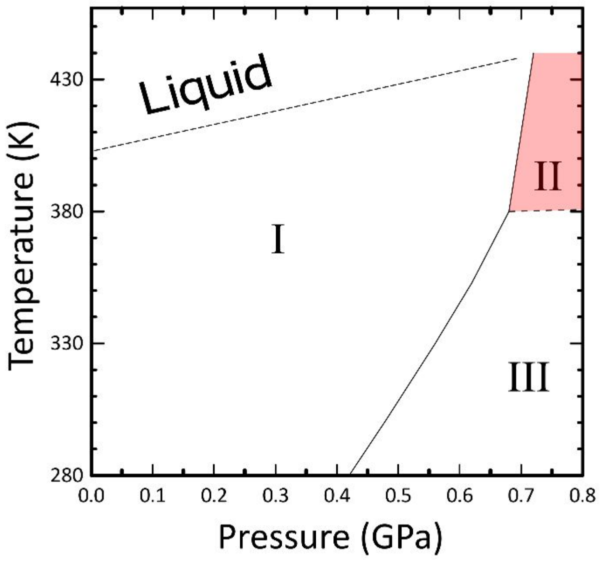

Figure S2 Phase diagram of urea obtained in Volumetric experiment with no pressure transmitting medium by P.W. Bridgman.[3] Postulated phase II (marked red) was not confirmed by other methods.[6,9]

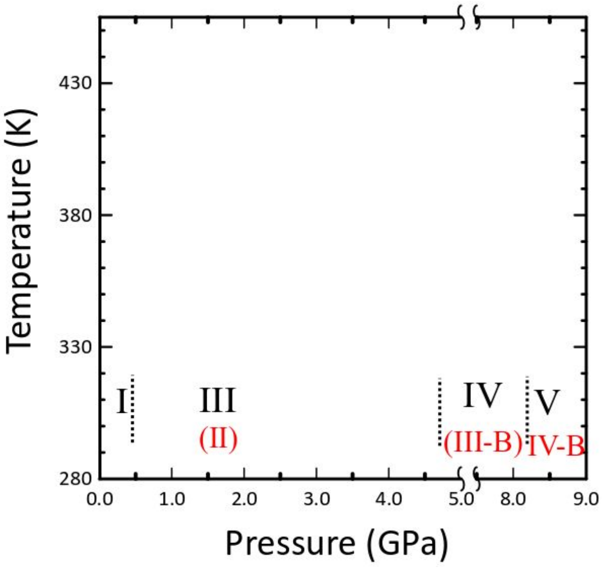

Figure S3 Phase diagram of urea from Lamelas *et.al* by Raman and derived from X-ray diffraction on the sample compressed in Ar.[4]

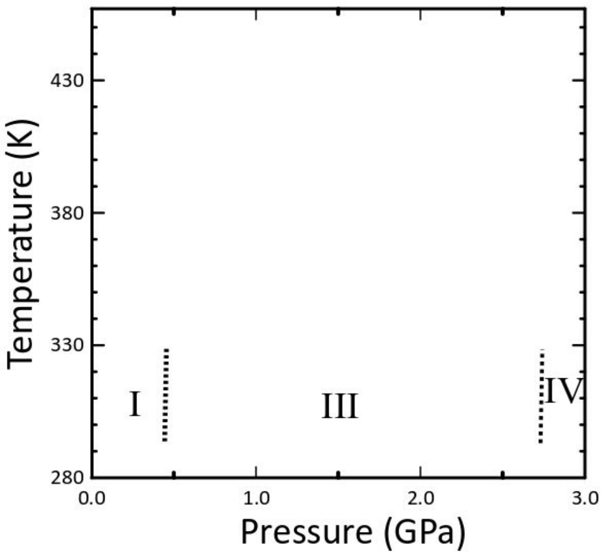

Figure S4 Phase boundaries of urea at 296 K based on the sample recrystallizations under pressure from the solutions in water (phases I and III) and in the methanol:ethanol:water mixture (phases III and V).[5,6]

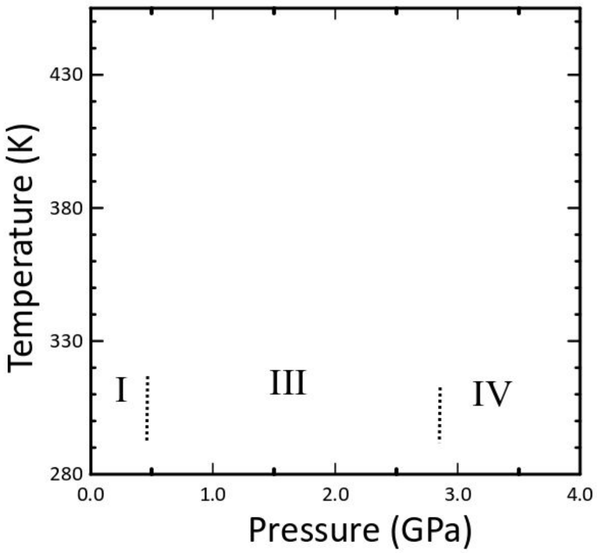

Figure S5 Measurements performed by neutron diffraction for deuterated urea compressed in deuterium at room temperature by Donnelly *et.al.*[7]

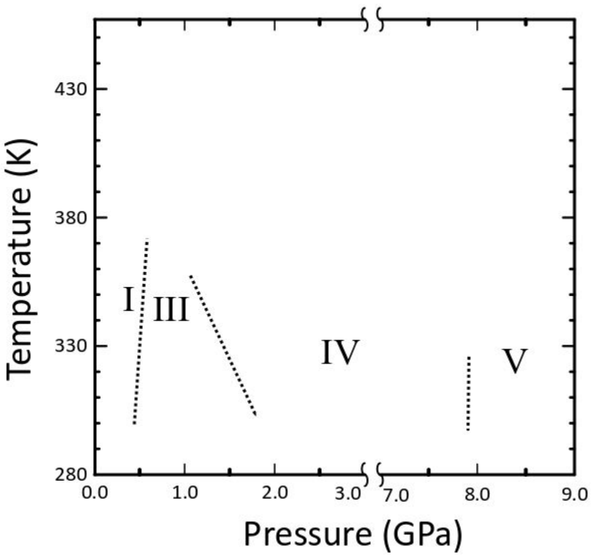

Figure S6 Phase diagram of pure urea by FTIR from Dziubek *et.al.*[8,9]

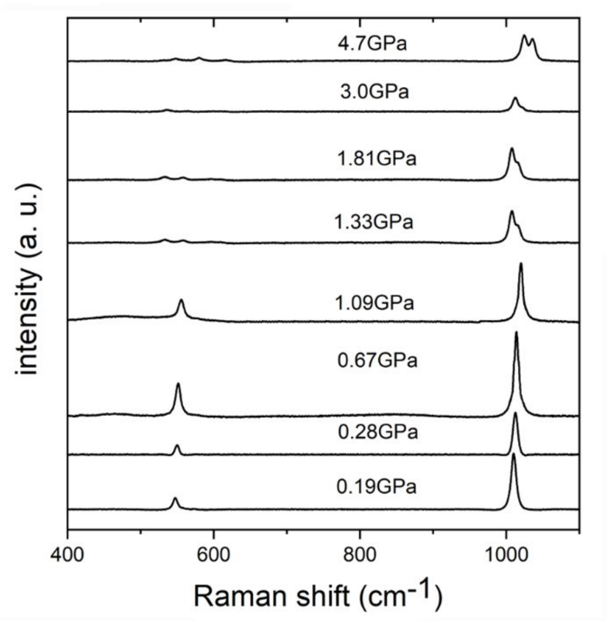

Figure S7 Raman spectra of urea in immersion oil up to 4.7 GPa, the intensities of spectra are normalized to allow comparison all mode.

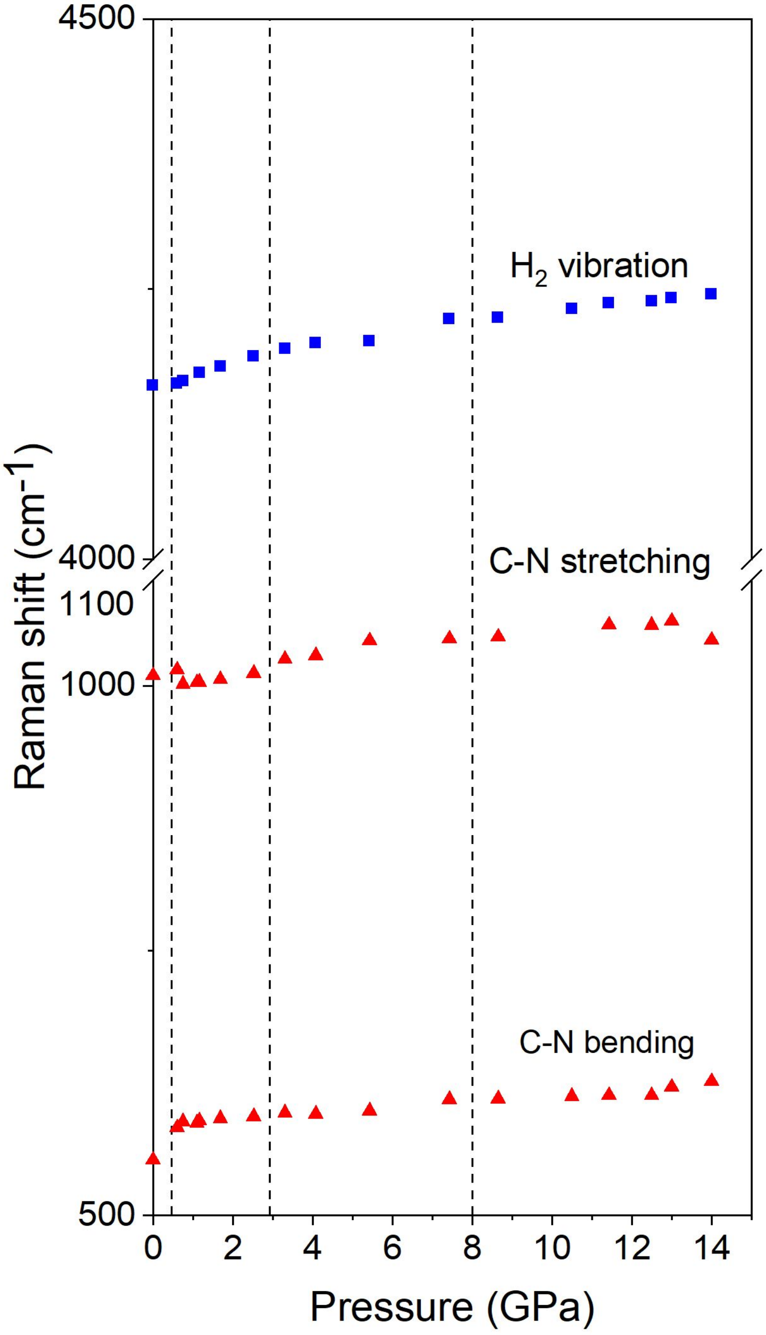

Figure S8 Hydrogen (Q_1-branch) and urea frequencies as a function of pressure up to 14.0 GPa. The hydrogen frequencies are indicated in blue and those of urea in red.

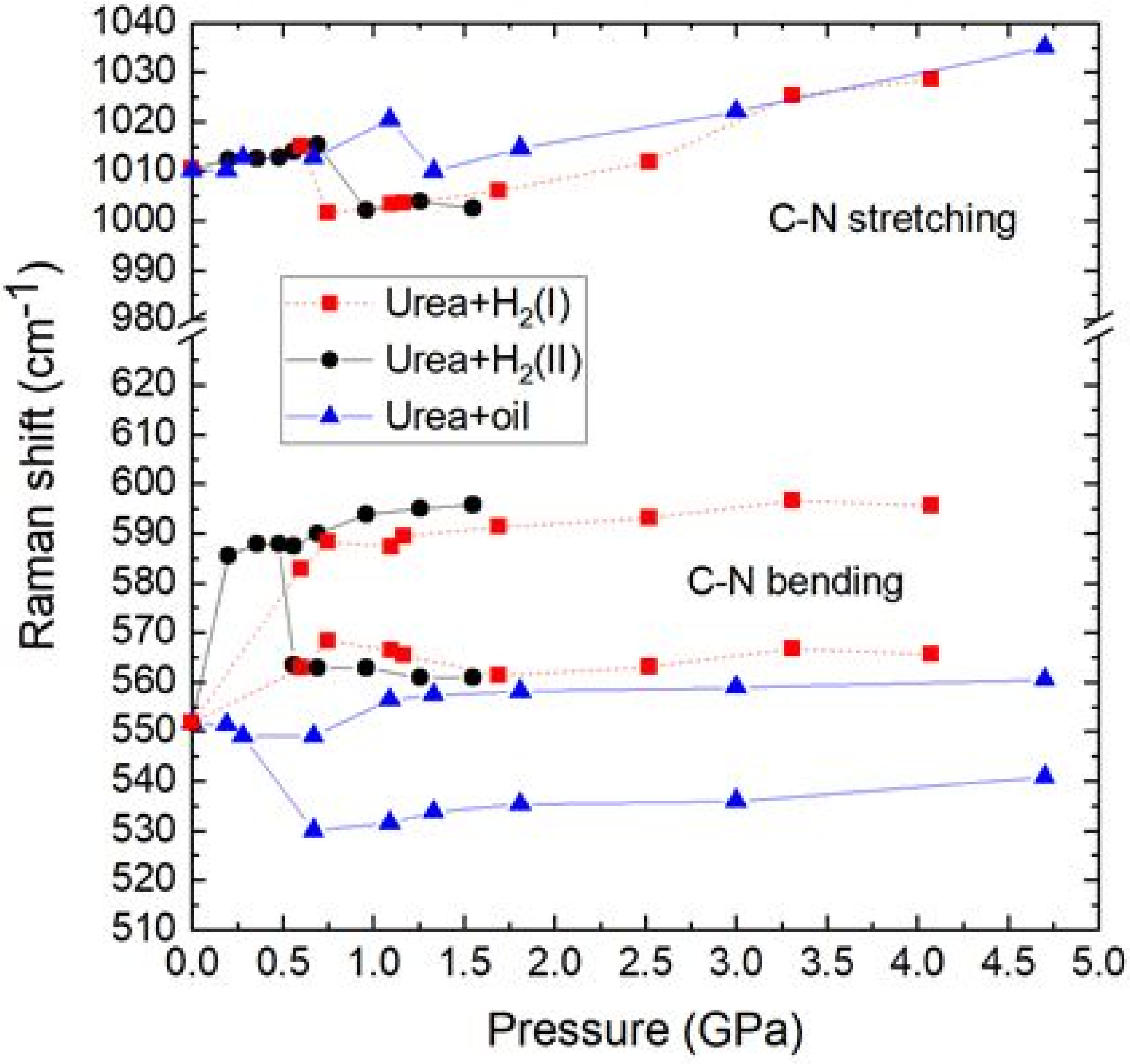

Figure S9. Raman shifts frequencies as a function of pressure measured for urea powder compressed in oil and in hydrogen (see the legend). The lines joining the points are for guiding the eye only.

References

(1) Paliwoda, D.; Kowalska, K.; Hanfland, M.; Katrusiak, A. U-Turn Compression to a New Isostructural Ferrocene Phase. *J. Phys. Chem. Lett.* **2013**, *23*, 4032–4037, DOI: 10.1021/jz402254b

(2) Budzianowski, A.; Katrusiak, A. Pressure-Frozen Benzene I Revisited. *Acta Crystallogr B* **2006**, ***62***, 94–101. DOI: 10.1107/S010876810503747X

(3) P. W. Bridgman, *Proc. Am. Acad. Arts Sci.* 1916, **52**, 91–187.

(4) Lamelas, F. J.; Dreger, Z. A.; Gupta Y. M. Raman and X-Ray Scattering Studies of High-Pressure Phases of Urea. *J. Phys. Chem. B* **2005**, *109*, 8206–8215, DOI: 10.1021/jp040760m.

(5) Olejniczak, A.; Ostrowska, K.; Katrusiak, A. H-Bond Breaking in High-Pressure Urea. *J. Phys. Chem. C* **2009**, *35*, 15761–15767, DOI: 10.1021/jp904942c

(6) Roszak, K.; Katrusiak, A. Giant Anomalous Strain between High-Pressure Phases and the Mesomers of Urea. *J. Phys. Chem. C* **2017**, *121*, 778–784, DOI: 10.1021/acs.jpcc.6b11454

(7) Donnelly, M.; Bull, C. L.; Husband, R. J.; Frantzana, A. D.; Klotz, S.; Loveday, J. S. Urea and Deuterium Mixtures at High Pressures. *J. Chem. Phys.* **2015**, *142*, 124503–124507, DOI:10.1063/1.4915523

(8) Weber, H. P.; Marshall, W. G.; Dmitriev. V. High-Pressure Polymorphism in Deuterated Urea *Acta Crystallogr., Sect. A*: *Found. Crystallogr*. **2002**, *58*, 174–185, DOI: 10.1107/S0108767302091985

(9) Dziubek, K.; Citroni, M.; Fanetti, S.; Cairns*,* A. B.; Bini, R. High-Pressure High-Temperature Structural Properties of Urea. *J. Phys. Chem. C* **2017**, *121*, 2380–2387, DOI: 10.1021/acs.jpcc.6b11059
